# A bibliometric analysis: Ca^2+^ fluxes and inflammatory phenotyping by flow cytometry in peripheral blood mononuclear cells

**DOI:** 10.3389/fimmu.2023.1272809

**Published:** 2023-10-13

**Authors:** Camille Brun, Lucie Chalet, Florentin Moulin, Thomas Bochaton, Sylvie Ducreux, Melanie Paillard, Claire Crola Da Silva

**Affiliations:** ^1^ Univ Lyon, CarMeN Laboratory, INSERM, INRA, INSA Lyon, Université Claude Bernard Lyon 1, Bron, France; ^2^ Olea Medical, La Ciotat, France; ^3^ Hospices Civils de Lyon, Hôpital Louis Pradel, Services D’explorations Fonctionnelles Cardiovasculaires et CIC de Lyon, Lyon, France

**Keywords:** flow cytometry, B cells, T cells, Ca^2+^ signaling, kinetics, immune cells, inflammation

## Abstract

**Background:**

The immune system, composed of organs, tissues, cells, and proteins, is the key to protecting the body from external biological attacks and inflammation. The latter occurs in several pathologies, such as cancers, type 1 diabetes, and human immunodeficiency virus infection. Immunophenotyping by flow cytometry is the method of choice for diagnosing these pathologies. Under inflammatory conditions, the peripheral blood mononuclear cells (PBMCs) are partially activated and generate intracellular pathways involving Ca^2+^-dependent signaling cascades leading to transcription factor expression. Ca^2+^ signaling is typically studied by microscopy in cell lines but can present some limitations to explore human PBMCs, where flow cytometry can be a good alternative.

**Objective:**

In this review, we dived into the research field of inflammation and Ca^2+^ signaling in PBMCs. We aimed to investigate the structure and evolution of this field in a physio-pathological context, and then we focused our review on flow cytometry analysis of Ca^2+^ fluxes in PBMCs.

**Methods:**

From 1984 to 2022, 3865 articles on inflammation and Ca^2+^ signaling in PBMCs were published, according to The Clarivate Web of Science (WOS) database used in this review. A bibliometric study was designed for this collection and consisted of a co-citation and bibliographic coupling analysis.

**Results:**

The co-citation analysis was performed on 133 articles: 4 clusters highlighted the global context of Ca^2+^ homeostasis, including chemical probe development, identification of the leading players in Ca^2+^ signaling, and the link with chemokine production in immune cell function. Next, the bibliographic coupling analysis combined 998 articles in 8 clusters. This analysis outlined the mechanisms of PBMC activation, from signal integration to cellular response. Further explorations of the bibliographic coupling network, focusing on flow cytometry, revealed 21 articles measuring cytosolic Ca^2+^ in PBMCs, with only 5 since 2016. This final query showed that Ca^2+^ signaling analysis in human PBMCs using flow cytometry is still underdeveloped and investigates mainly the cytosolic Ca^2+^ compartment.

**Conclusion:**

Our review uncovers remaining knowledge gaps of intracellular players involved in Ca^2+^ signaling in PBMCs, such as reticulum and mitochondria, and presents flow cytometry as a solid option to supplement gold-standard microscopy studies.

## 1 Introduction

Blood samples are routinely used in clinics for disease diagnosis or prognosis. The main components of blood are plasma and leukocytes. The immunophenotyping of leukocytes, notably the peripheral blood mononuclear cells (PBMCs), has emerged as an essential tool for medical research. This diagnostic tool is widespread in hematology, cancerology, and neurology fields.

For clinical diagnosis, multiparametric phenotyping is typically achieved using flow cytometry. This method achieves 10000 to 40000 cell reads per second on average with the most recent cytometers, enabling statistical robustness compared to microscopy imaging, high sensitivity and specificity to detect the most under-represented subpopulations, and fast diagnosis (usually under 48 hours) (1, 2). Diagnosis is based on detecting PBMC-specific membrane glycoproteins called “cluster of differentiation” (CD), commonly associated with their immune function and cell subpopulation (3). The simultaneous expression of multiple CD markers serves as a cellular signature comparable to an “identity card.” PBMCs can easily be isolated from blood sampling followed by centrifugation. Therefore, PBMCs have recently emerged as complementary biomarkers to stratify patients in several pathologies by characterizing their immunophenotypes. For instance, monitoring the level of CD4^+^ T cells in Human Immunodeficiency Virus (HIV) infections is crucial to monitoring disease progression (4). Flow cytometry has also become essential to study the activation of basophilic cells in the presence of a given allergen (5) and can even be used to diagnose acute leukemia (6). In fact, flow cytometry is extensively used in several clinical applications notably in clinical practice. Indeed, flow cytometry is largely used to characterize diseases such as malignancies (leukemia, lymphoma (7, 8)), infectious diseases (9) and degenerative diseases (10, 11) through immunophenotyping. More recently, Obasanmi et al. studied PBMC cytokine production levels in patients with type 1 diabetes and diabetic retinopathy. Their work revealed that PBMCs from diabetic patients have a specifically enhanced interleukin-10 (IL-10) and interleukin-6 (IL-6) releases, associated with increased interleukin-17A (IL-17A) production from myeloid cells and impaired CD3^+^ T cell-induced interferon-gamma (IFN-γ) production (12). Flow cytometry is recognized as the method of choice for immunophenotyping based on this non-exhaustive list of examples.

Under inflammatory conditions, PBMCs are partially activated by T or B cell membrane receptors (TCR or BCR, respectively) and Fc receptors for monocytes and macrophages. Their activation through these receptors involves a Ca^2+^-dependent signaling cascade, starting from the ligand binding on the receptor through intracellular pathways up to the regulation of gene expression. More precisely, membrane receptor activation triggers a transitory Ca^2+^ release from internal stores, further leading to a store-operated Ca^2+^ entry (SOCE), raising the intracellular Ca^2+^ concentration (13, 14), which in turn, regulates several processes such as proliferation, phagocytosis, chemotaxis, and cytokine secretion (15). Thus, Ca^2+^ is considered as a key regulator of the immune cell. In 1994, Partiseti et al. reported an altered Ca^2+^ influx following TCR aggregation in native T cells in immunodeficient patients, visualized with microscopy and electrophysiological recordings (16). So far, microscopy is the most common method to identify and analyze Ca^2+^ fluxes in cell line models. It offers the advantage of visualizing the intracellular architecture of the cell. However, the statistical power brought by the number of events analyzed, the high acquisition rate, and the possibility of exploring a more significant number of parameters simultaneously on the same sample all favor the use of flow cytometry. Consequently, it represents a powerful alternative to assess Ca^2+^ fluxes, but still remains underused. It has been shown that alteration or modulation of calcium signaling can impact immune cell function in some pathologies (17, 18). To identify existing protocols for analyzing calcium signaling, we proposed this bibliometric review questioning the role of flow cytometry in studying Ca^2+^ signaling, specifically in peripheral blood mononuclear cells. To this end, we first outlined the inflammatory and Ca^2+^ research field and then focused on Ca^2+^ homeostasis studies performed by flow cytometry on PBMCs.

## 2 Materials and methods

### 2.1 Bibliometric analysis

**Figure 1:**
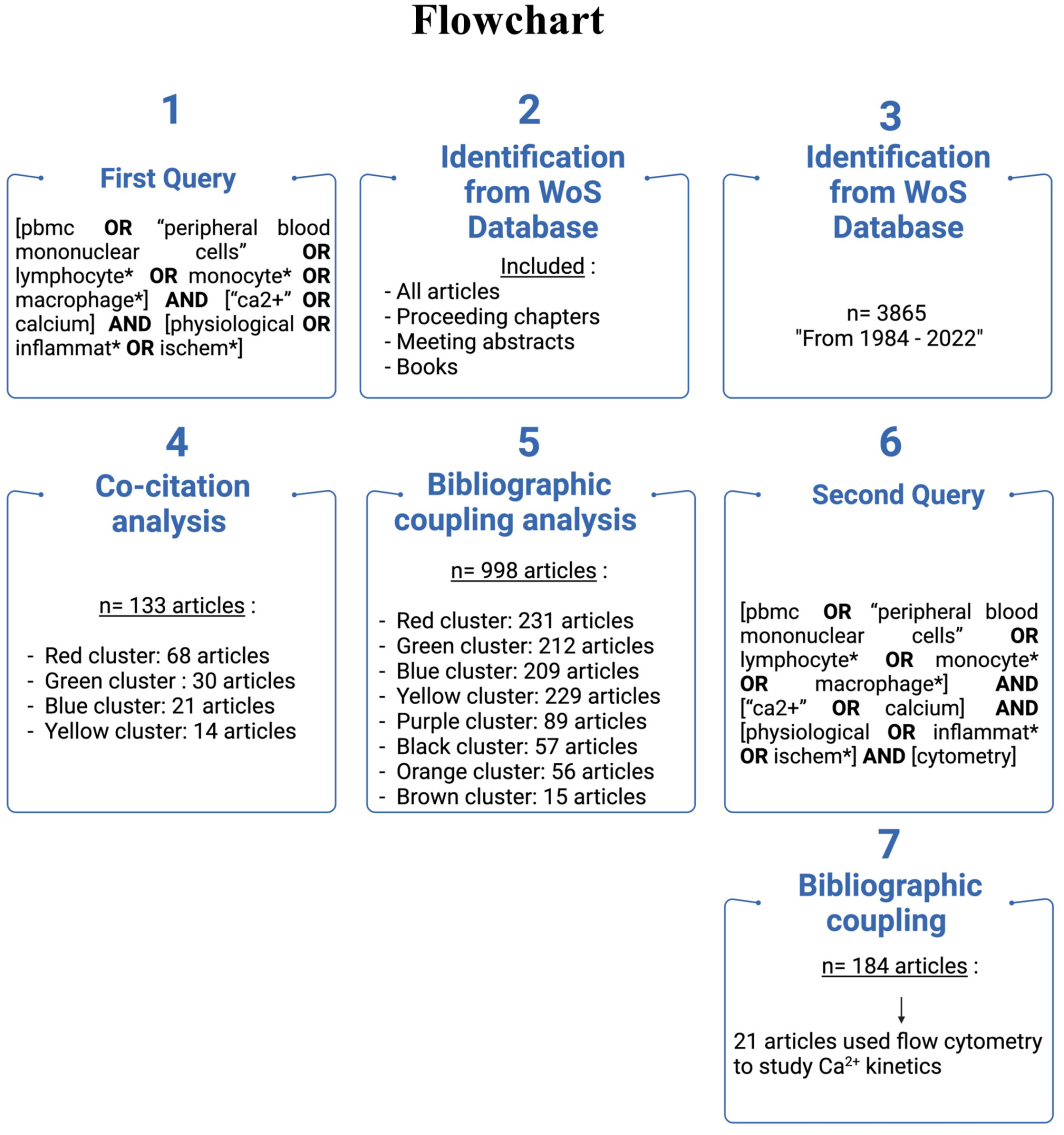
Flowchart showing the methodology to identify, filter and analyze papers.

The bibliometric analysis is suited when the scope of the review is broad and the dataset is too large for manual review. This analysis presents the intellectual structure and emerging trends of a research topic. The study was designed following the guidelines of Donthu et al. and the methodology of Chalet et al. (19, 20). The flowchart depicted in **Figure 1** summarizes the methodology used to highlight the literature selection.

### 2.2 Scope and aim of the analysis

We aimed to determine the research structure and evolution of inflammatory and Ca^2+^ signaling in PBMCs in a physio-pathological context. Based on these first results, we further explored the development of Ca^2+^ analysis by flow cytometry.

### 2.3 Definition and selection of bibliometric analysis techniques

The structure and dynamics of the scholar knowledge were assessed and represented using science maps. To provide an overview of our research topic and to explore the emerging trends, co-citation analysis and bibliographic coupling were carried out.

- The co-citation analysis determines the relationships among cited publications, highlighting the most influential themes.
- The bibliographic coupling forms thematic clusters.

### 2.4 Collecting scientific literature data

Clarivate Web of Science^©^ was used to collect data (Copyright Clarivate 2022WoS). It is a selective, structured, and balanced database with complete citation links and enhanced metadata. It can inform about citation indexes representing the connections between scholarly research articles in globally significant journals, books, and proceedings chapters. Exported data included the complete set of references enabling in-depth analysis of the intellectual structure.

We applied the following query to article titles and abstracts:

[pbmc OR “peripheral blood mononuclear cells” OR lymphocyte OR monocyte* OR macrophage*] AND [“Ca^2+^” OR calcium] AND [physiological OR inflammat* OR ischem*]

We included all articles, proceeding chapters, meeting abstracts, and books. The query ranged from 1984 to 2022, and 3865 articles were collected. All data were exported from the WoS database on July 22, 2022.

Further analysis of the field, focusing on the evolution of Ca^2+^ analysis by flow cytometry, was carried out based on the following query applied to abstracts and titles:

[pbmc OR “peripheral blood mononuclear cells” OR lymphocyte* OR monocyte* OR macrophage*] AND [“Ca^2+^” OR calcium] AND [physiological OR inflammat* OR ischem*] AND [cytometry]

We included all articles, book chapters, and meeting abstracts. The query ranged from 1993 to 2022, and 185 articles were obtained.

### 2.5 Network generation and display

VOS viewer was used to display bibliometric networks represented through mapping and clustering (21) to perform the analysis.

### 2.6 Co-citation method

Two articles are co-cited when they appear together in the reference list of another publication. Frequently co-cited documents in a corpus represent its knowledge foundations. This technique highlights influential publications and unveils the structure of the research field. The co-citation was conducted in VOS viewer using reference analysis. The results were displayed as network visualization. The minimum number of co-cited references was fixed to 20 to obtain the most influential articles. 133 co-cited documents were included in our analysis and mapping, and were clustered and displayed on the VOS viewer. Included references grouped by clusters are displayed in **Supplementary Figure S1**. To determine the main subject of each cluster, we focused our analysis on the most significant publications in each cluster, i.e., in the 3^rd^ quartile.

### 2.7 Bibliographic coupling method

The bibliographic coupling method considers publications that share common references as an indication of similarities in content. This analysis provides a clustered visualization of the field in themes and includes recent and niche publications. The bibliographic coupling analysis was obtained with VOS viewer, with coupling analysis of references. The results were displayed as network visualization. This bibliographic coupling was carried out to determine the knowledge in the field of Ca^2+^ and inflammation in PBMCs. Articles from 1990 to 2022 were included, and 998 were grouped in clusters. Cluster constituents are detailed in **Supplementary Figure S2**.

The exploration of the clusters was performed on the highest total link strength articles, corresponding to the link of an item with other items in a network. To get an overview of the dominant theme of each cluster, the references with a total link strength equal to or higher than the 3^rd^ quartile of the full scores in their cluster were selected for a thorough analysis.

### 2.8 Limitations of bibliographic coupling analysis

The term “inflammat*” covers a large field of research, including several pathologies. Thus, the most influential articles in our “inflammatory” cluster discuss chemokines in HIV. The abbreviation T cells or B cells were not included in the query.

## 3 Results

### 3.1 General information: Trend of publication

**Figure 2:**
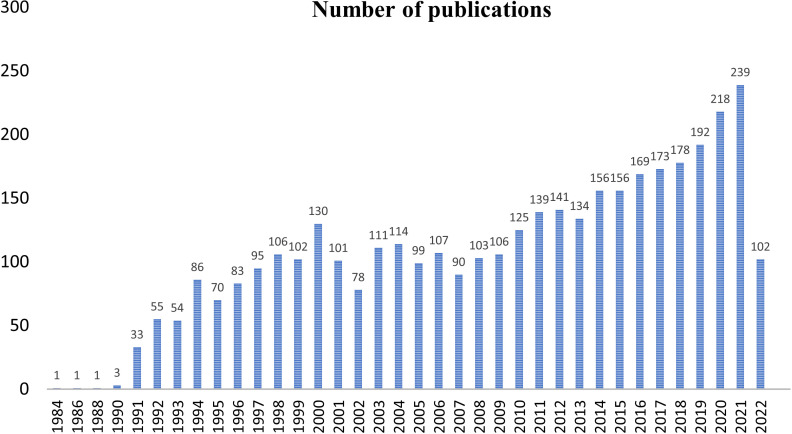
Annual number of publications worldwide from 1984 to 2022.

**Figure 3:**
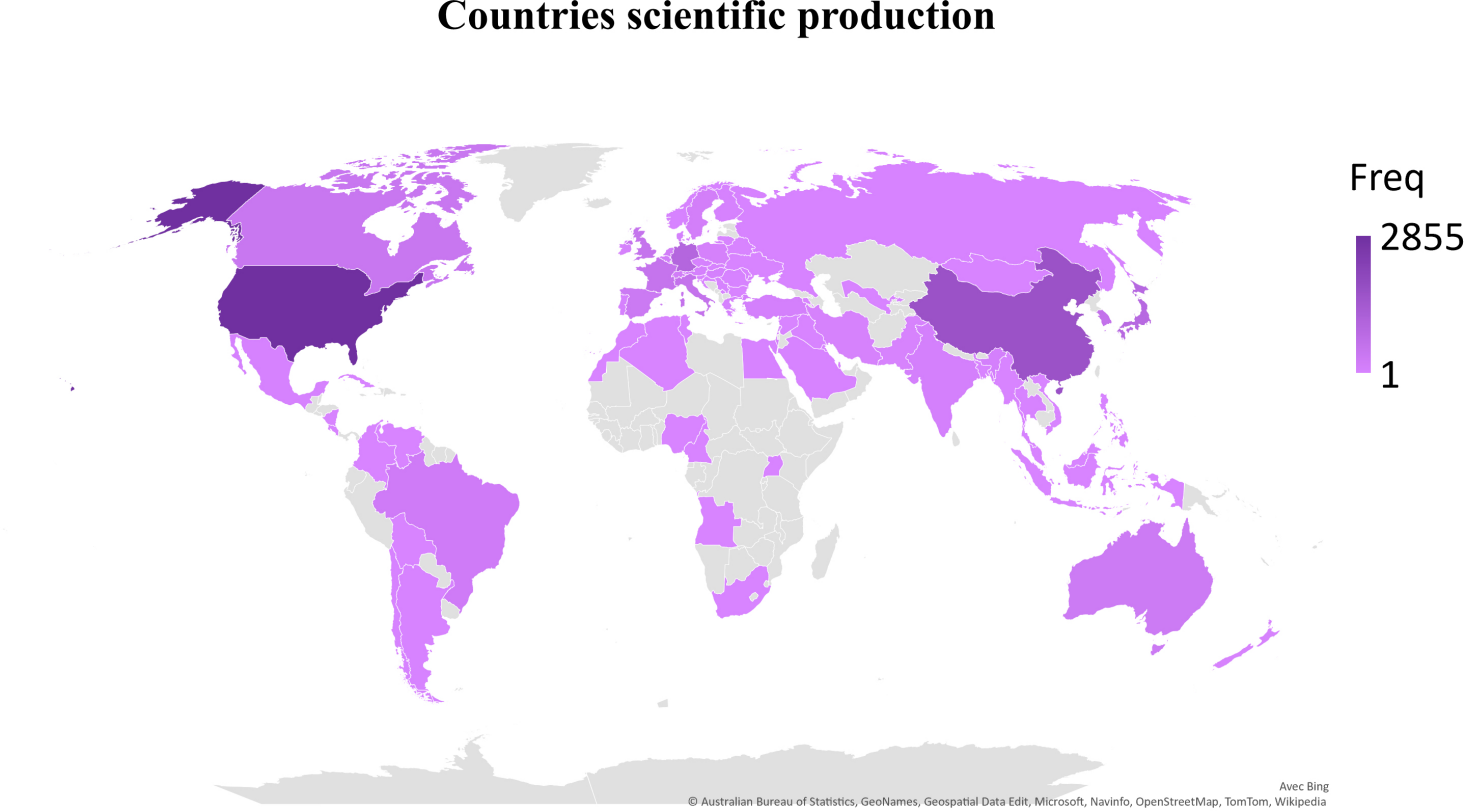
Scientific production by country, displayed by the total number of publications over the query time (Freq).

**Figure 4:**
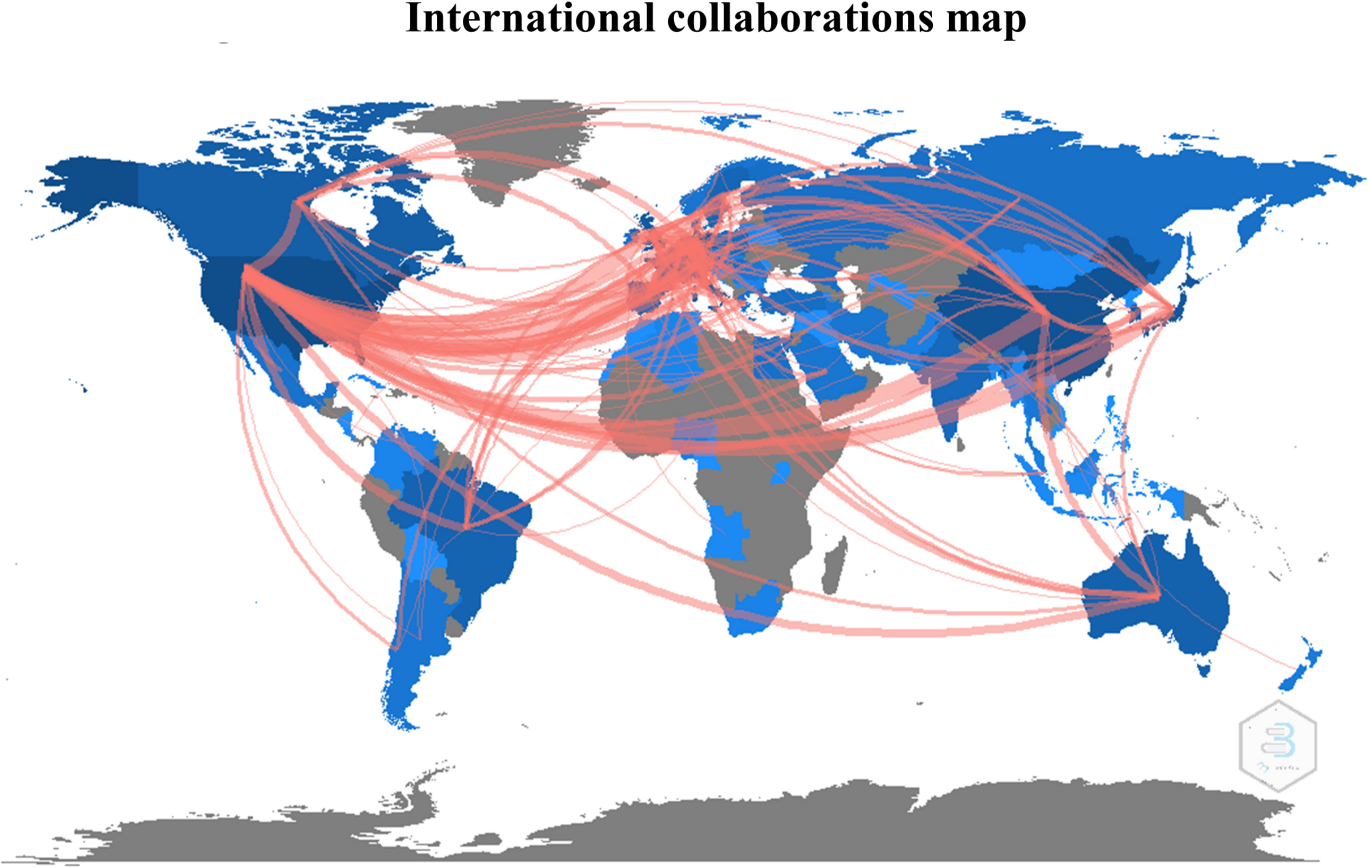
International collaborations map, represented by the number of collaborative studies between countries.

The first query aimed to overview Ca^2+^ and inflammation research in immune cells in the current literature. In total, 3865 articles were published between 1984 and 2022. An average of 90 publications per year between 1990 and 2010 were reported. Over the next ten years, publications increased strongly, up to 2-fold from 2010 to 2021. Analysis was done until July 2022, which explains the lowest number of publications for this year (**Figure 2**). Most of them were published in the United States of America and China and, to a lesser extent, in Europe, Canada, and South America (**Figure 3**). However, the number of collaborations between all these countries was important. Collaborations for these articles were made mainly between the USA, European countries, and China, indicating a worldwide interest in the Ca^2+^ and inflammation topic in immune cells (**Figure 4**).

### 3.2 Co-citation analysis

**Figure 5:**
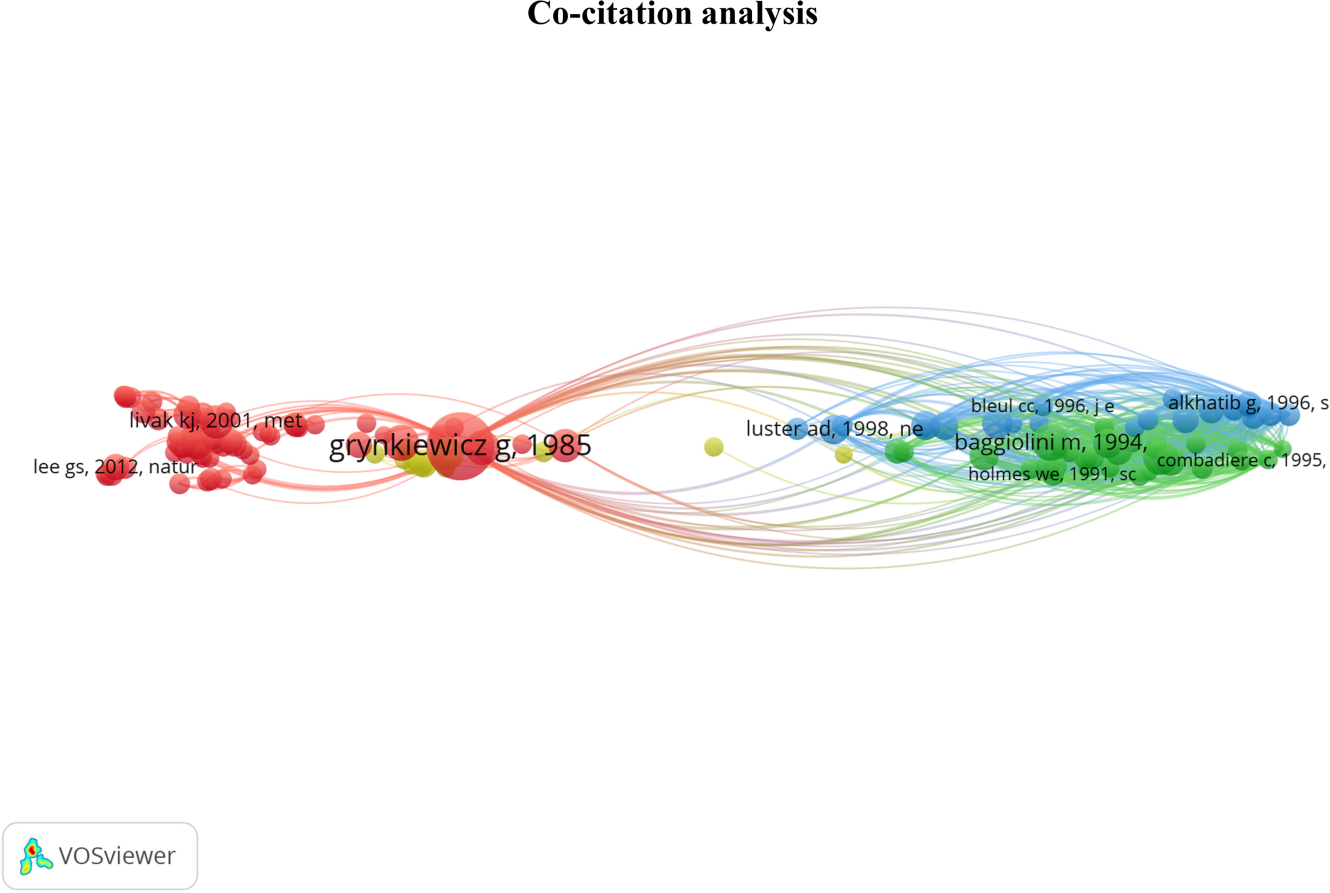
Co-citation network providing an overview of the most contributing publications in the Ca^2+^ and inflammation context. Red cluster [1976 - 2006]: Development of Ca^2+^ probes, Ca^2+^ signaling, and channels identified in the plasma membrane. Yellow cluster [1987 - 1997]: intracellular pathways & calcium-binding proteins; Green cluster [1991 - 1995]: chemokine receptors; Blue cluster [1995 - 2006]: RANTES chemokines in HIV.

The co-citation analysis provides an overview of the most substantial contributions to the field by measuring the frequency of two articles being cited together in a scientific literature corpus (19). The analysis was done on the 133 most co-cited documents grouped into 4 clusters. This threshold highlights the most influential articles. The cluster denomination was based on articles from the previously described selection method. The co-citation network is displayed in **Figure 5**. The cluster position provides information about the relationship between topics. We observed the closeness of the red and yellow clusters and their remote connection to the blue and green clusters. The content of each cluster will be further developed to provide interpretation resources on the relations between these groups.

In the red cluster, we observed two groups; one has a central position and is related to all the other clusters, i.e., yellow, green, and blue clusters. In the red cluster, 17 out of 68 publications were in the 3^rd^ quartile, ranging from 1976 to 2012. The red cluster relates to Ca^2+^ signaling and channels in the plasma membrane. Most publications are focused on the Stromal Interaction Molecule (STIM), a Ca^2+^ sensor essential for the SOCE through its binding to ORAI1 (Calcium Release-Activated Calcium Modulator 1). ORAI proteins are STIM-binding partners that form the channel pore in the plasma membrane (22, 23). These channels represent the main pathway of Ca^2+^ influx in T- and B-cells and promote the immune response by partly activating the transcription factor nuclear factor of activated T-cells (NFAT) (14, 24, 25). The study by Grynkiewicz et al., published in 1985 (26), appears in a central position. This article deals with a new generation of highly fluorescent indicators to study the physiological role of cytosolic free Ca^2+^ concentration, with greatly improved fluorescence properties. Most articles in the second part of the red cluster use the probes described in Grynkiewicz’s article, notably Fura2-AM (27–29). Therefore, this cluster highlights the fundamental knowledge of Ca^2+^ signaling in PBMCs.

Near the red cluster, the yellow cluster includes 3 out of 14 publications in the 3^rd^ quartile ranging from 1987 to 1991. They refer to calcium-binding proteins (CaBPs), such as the S100 protein. S100 was reported to be associated with specific stages of monocyte differentiation (30, 31). S100 function is still unclear, but some evidence suggests that macrophages infiltrated during inflammation express the myeloid-related protein 8 (MRP8) and the myeloid-related protein 14 (MRP-14) members of the S100 protein family in rheumatoid arthritis pathology (32). These two clusters related to the Ca^2+^ topic support the critical role of Ca^2+^ signaling in immune cell function.

The last two clusters, green and blue, are located on the opposite side of the maps and share close positions and content. No temporal evolution was observed in these two clusters, since all the articles were published in the 90s (33–36). The green cluster contains 7 out of 30 articles in the 3^rd^ quartile, ranging from 1991 to 1995, which refer to chemokine receptors involved in inflammation. In its vicinity, the blue cluster with 5 out of 21 articles in the 3^rd^ quartile, from 1995 to 1996, focuses on chemokines, especially the regulated-on activation, normal T cell expressed and secreted (RANTES) or chemokine ligand 5 (CCL5) axis in HIV infections. Indeed, RANTES research was achieved at the time of the outbreak emergence of HIV at the beginning of the 1980s, instigating extensive research on this chemokine.

To conclude, the co-citation analysis supports the crucial role of Ca^2+^ and chemokine signaling in the immune cell function, which depends on signal integration, stress, chemical environment, and inflammation, key features of several pathologies. More precisely, all these parameters lead to modulation and cell activation by intracellular pathways, activating CaBPs and partially leading to their translocation to the nucleus. The latter activates Ca^2+^-dependent transcription factors that control PBMC functions such as proliferation, differentiation, and cytokine production.

### 3.3 Bibliographic coupling analysis

**Figure 6:**
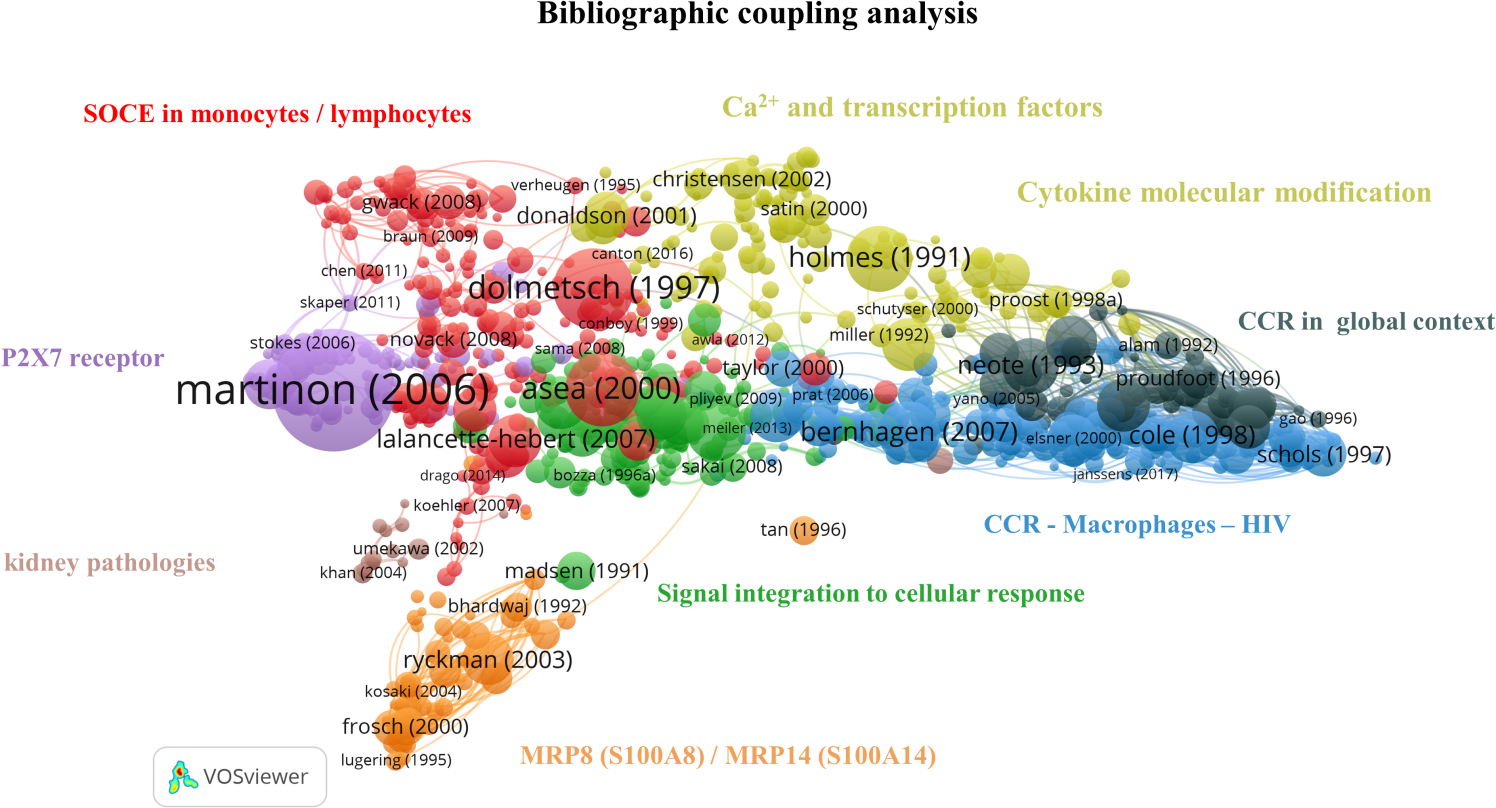
Bibliographic coupling network: Ca^2+^ and inflammation in PBMCs [1990-2023]. The red cluster represents the CRAC channel in lymphocytes and monocytes. The purple cluster focuses on the P2X7 purinergic receptor in macrophages. The orange cluster refers to the specific calcium-binding protein MRP8 (S100A8)/MRP14 (S100A14). The green cluster covers the cascade from signal integration to cellular response. The yellow cluster relates to Ca^2+^ signaling, transcription factors and cytokine molecular modifications. The black cluster targets chemokine receptors, the blue cluster accounts for chemokine receptors in HIV infections, and the brown cluster focuses on kidney pathologies.

Bibliographic coupling is based on the idea that two publications sharing common references have similar content. This analysis enables the formation of thematic clusters in the literature corpus obtained with our query. Consequently, recent niche publications may appear in our analysis. This section aimed to determine what drove the calcium and inflammation research field on PBMCs over the last three decades. The analysis was done on 998 articles, and 8 clusters were identified (**Figure 6**). The large number of articles and the link between them make the interpretation complex; a preliminary exploration of the clusters showed a large representation of pathologies involving an immune response, and the in-depth analysis of the clusters provided further knowledge on the field structure.

The red cluster contains 57 out of 232 articles in the 3^rd^ quartile and refers to the SOCE. This cluster can be further divided into subgroups related to lymphocytes and macrophages, respectively.

We identified articles about the calcium release-activated channel (CRAC) components in the lymphocyte cluster. In 2005 – 2006, both STIM1 Ca^2+^ sensor and ORAI1 channel-forming protein were determined by Feske et al. The CRAC composition depends on cell type, localization, and activation state, but ORAI1 is the dominant channel member in several immune cells, mainly in neutrophils and mast cells (37–39). In physiological conditions, the CRAC channel is activated by inositol 1,4,5 phosphate (IP_3_) binding to IP_3_ receptors (IP_3_R) in the endoplasmic reticulum (ER) membrane following TCR or BCR activation. IP_3_ leads to IP3R opening, resulting in Ca^2+^ release from the ER into the cytosol. The resulting ER Ca^2+^ decrease induces a conformational change of STIM1, which binds to ORAI1 channels to open its pore in the plasma membrane. The importance of Ca^2+^ influx through plasma membrane channels in T cells was characterized by studying the Ca^2+^ conductance triggered by the TCR. An absence of this current was demonstrated in human T cells from patients with immunodeficiency diseases (16). Missense mutations in ORAI1 affect the channel function and the subsequent T-cell function leading to severe immunodeficient phenotypes (40, 41).

In non-excitable cells such as lymphocytes, potassium (K^+^) channels were identified in 1983 by Matteson and Deutschand in 1984 by DeCoursey et al. The latter publication shows their implication in functional processes such as mitogenesis (42). In T-lymphocytes, the engagement of the TCR/CD3 complex upon antigen binding leads to the increase of intracellular Ca^2+^. This Ca^2+^ influx is maintained by K^+^ channels through K^+^ release outside the cells, preserving the electrochemical potential gradient. Panyi et al. reported that the interleukin-2 (IL-2) production and cell proliferation in T cells are partly mediated by K^+^ channels (43). The inhibition of the K^+^ channel, named Kv1.3, inhibits T-cell activation, calcium signaling, cytokine production, and cell proliferation (44).

STIM1 and ORAI proteins were also identified in phagocytic immune cells, notably in macrophages, as demonstrated in papers in the related sub-clusters (38). There are several phenotypes of macrophages: the pro-inflammatory one, also known as classical monocytes (M1), with an important phagocytosis function; and the anti-inflammatory one as non-classical monocytes (M2). Macrophage plasticity is essential for innate immunity since macrophages can switch their phenotype according to their chemical environment. Chauban et al. showed that ORAI1 significantly contributes to Ca^2+^ entry *in vitro* using non-differentiated macrophages (M0). In contrast, *in vitro* M1 polarization induced by IFN_ɣ_ is associated with the recruitment of the transient receptor potential cation channel 1 (TRPC1) to enhance Ca^2+^ entry, leading to high expression of inflammatory genes (45).

Additionally, this subcluster references the monovalent cation channel transient receptor potential melastatin (TRPM), involved in the physiological response in some immune cells, i.e., monocytes and macrophages, through intracellular Ca^2+^ level regulation. High intracellular Ca^2+^ concentration leads to TRPM4 opening, regulating Na^+^ entry and Ca^2+^ efflux. Indeed, TRPM4 works in concert with the CRAC channel to achieve this regulation. Serafini et al. studied TRPM4 deletion in a sepsis mouse model. They observed an altered function in the absence of TRPM4 through a decrease in phagocytosis and an increased pro-inflammatory cytokine production, leading to an alteration of macrophage function affecting the mouse survival (46).

In summary, the red cluster highlights the central role of Ca^2+^ signaling in the immune function of macrophages and lymphocytes. It therefore represents the largest cluster in our query, gathering around 23% of the articles in the bibliographic coupling map.

Close to the red cluster, the main topic of the purple cluster is centered on purinergic receptors, such as P2X7, and includes 22/89 articles in the 3^rd^ quartile, from 2003 to 2016. It is well known that Ca^2+^ signaling enhances mitochondrial adenosine triphosphate (ATP) production in activated T cells (14). ATP is then exported outside T cells through the Pannexin 1 channel and activates P2X7, which causes further Ca^2+^ entry (14). P2X7 receptors are expressed on mast cells, lymphocytes, erythrocytes, fibroblasts, and peripheral macrophages. In monocytes/macrophages, P2X7 receptor activation leads to interleukin production, notably interleukin 1-bêta (IL-1β) as a pro-inflammatory factor (47).

The orange cluster contains 14/57 publications in the 3^rd^ quartile and is localized in the network periphery. It refers to the specific CaBP S100 (48), an inflammatory marker, thus explaining its remote connection to the other inflammation-themed clusters (49).

In the green cluster, 27 articles cover the role of intracellular pathways in immune cells, including Ca^2+^ fluxes, receptors, and transcription factors such as nuclear factor kappa B (NF-κB) (50–52). The remaining 7 publications focus on cytokines, key soluble elements of signal integration in cellular stress (53–59). Therefore, this cluster gives an overview of immune cell signal integration from the cytokine binding to its receptor up to the gene expression, explaining its central position on this bibliographic coupling map.

The yellow cluster is widely extended and includes 32/129 articles in the 3^rd^ quartile. A part of it is located close to the red cluster and covers articles related to Ca^2+^ signaling and transcription factors (60, 61). In contrast, the other part contains those related to cytokine molecular modification (62–64). This latter part is close to the black cluster that contains 14/58 publications in the 3^rd^ quartile and focuses on chemokine receptors (CCR) and their ligand. Chemokine receptors are G-protein-coupled-receptors serpentine receptors. Chemokines, a particular type of small cytokines, are known to contribute to the trafficking of leukocytes to the inflammation site through a signaling cascade. Depending on the environment, chemokines activate neutrophils to attract and activate monocytes, basophils, eosinophils, or lymphocytes (65). Several subclasses of chemokine receptors exist and are expressed constitutively or induced by inflammation (66). Moreover, some receptors can bind specific ligands or several chemokines. Thus, chemoattractants possess a crucial regulatory role in immunity and are involved in viral infection (67).

The blue cluster, with 53/210 articles in the 3^rd^ quartile, includes articles from 1997 to 2004, correlating with the HIV epidemic starting in 1981, and completes the preceding cluster by specifying the role of chemokine receptors in HIV infection. The HIV virion targets the cellular plasma membrane, and the fusion reaction occurs thanks to the viral envelope glycoprotein, which binds the CD4 surface marker (68). The virus uses the CXCR4 and CCR5 receptors for T cells and macrophages, respectively. The CXCR4 ligand is a natural ligand identified as stromal cell-derived factor 1 (SDF-1), also known as chemokine-12 (CXCL-12), and has the properties of a selective inhibitor of T cell tropism (69). Moreover, in 1995, Lusso et al. indicated that the CCL5 or RANTES cytokine, the macrophage inflammatory proteins 1α (MIP-1α/CCL3) and 1β (MIP-1β/CCL4) were all HIV-suppressive factors released by CD8^+^ T cells (70, 71).

The brown cluster focuses on kidney pathologies, especially on calcium oxalate crystal formation and on the role of monocytes-macrophages and the chemokine ligand 2 (CCL2), also known as monocyte chemoattractant protein 1 (MCP-1), in this pathology. This cluster contains 2/15 publications in the 3^rd^ quartile with two different topics. The first one investigates macrophage capacity to suppress renal crystal formation (72). At the same time, the second one refers to the CCL2 role in tubulointerstitial inflammation during kidney failure by inducing cytokine and adhesion molecule production (73). Of note, this cluster does not appear as the most relevant for the bibliometric coupling map analysis due to its low article number and its remote location and topic from other clusters mainly focused on Ca^2+^ signaling.

Altogether, this bibliographic coupling showed that Ca^2+^ signaling is widely studied in physiological conditions and several pathologies involving an immune response. Since intracellular Ca^2+^ signaling partly controls cytokine production, it was expected to see such a coupling between the Ca^2+^ signaling clusters and the inflammatory ones. Therefore, the resulting map aligns with our current field knowledge.

### 3.4 Ca^2+^ analysis by flow cytometry

Immunophenotyping using flow cytometry has become the gold standard method used in clinical (74) and fundamental research laboratories to characterize immune cells derived from patient blood samples. As the bibliographic coupling reveals, Ca^2+^ signaling is crucial in controlling immune cell functions. We next wanted to determine the evolution of flow cytometry-based studies to evaluate the Ca^2+^ kinetics in PBMCs over the last three decades. To perform this analysis, another query was defined to focus on flow cytometry, as specified in the “Materials and Methods” section. Ranging from 1993 to 2022, we obtained 184 articles. These articles were split into two flow cytometry usage categories: (1) for the phenotyping of immune cell profiles and (2) to analyze Ca^2+^ kinetics in PBMCs. We reported and focused our review on the 21 publications falling in the latter category (**Table 1**).

**Table 1:** Summary of the 21 articles using flow cytometry to analyze Ca^2+^ kinetics in PBMCs.

Articles	Cell type	Drugs	Ca^2+^ sensing probe	Date
Non-ratiometric probes				
Gauduchon et al. (75)	Human polymorphonuclear neutrophils	Panton-Valentine Leucocidin-derived protein	Fluo-3	2001
Kirchhoff et al. (76)	Human dendritic cells	Anaphylatoxins C5a and C3a	Fluo-3	2001
Princen et al. (77)	Lymphocyte and monocyte cell lines/Fresh PBMC	SDF-1	Fluo-3	2002
Heinemann et al. (78)	Human basophil cells	Anti-IgE, MCP-1, Eotoxin, MIP-1α, C5a and NGF	Fluo-3	2003
Nishizaki et al. (79)	Rat thymic lymphocytes	PbCl2 and Ca^2+^-dependant K^+^ channel activator (A23187)	Fluo-3	2003
Lamoureux et al. (80)	Human B lymphocytes	Cysteinyl-leukotrienes	Fluo-3	2006
Ceballos et al. (81)	Human immature dendritic cells	SPC	Fluo-3	2007
Chen et al. (82)	Human PBMC	Flagellin	Fluo-3	2013
Orbán et al. (83)	Human T lymphocytes	Phytohemagglutinin (PHA)	Fluo-3	2014
Gutzmer et al. (84)	Human monocytes derived dendritic cells	Histamine	Fluo-4	2002
Sun et al. (85)	BALB/c mice T lymphocytes	Concanavalin A	Fluo-4	2017
Tran et al. (86)	Human PBMC	Goitrin	Fluo-4	2018
Kushnir et al. (17)	Human B lymphocytes	Caffeine/4-CmC	Fluo-4	2018
Ratiometric probes				
Boltz et al. (87)	Human T lymphocytes	Margatoxin/Anti-CD3	Indo-1 and Fura-2	1994
Bates et al. (88)	Rabbit alveolar macrophages	Tannin	Indo-1	1995
Tárnok et al. (89)	Bovin alveolar macrophages	4 bromo - A23187	Indo-1 and Fluo-3	2001
Si et al. (90)	Human T lymphocytes	Phytohemagglutinin (PHA)	Fluo-4 and FuraRed	2005
Toldi et al. (91)	Human T lymphocytes	Margatoxin/triarylmethane/Phytohemagglutinin	Fluo-3 and FuraRed	2011
Toldi et al. (44)	Human T lymphocytes	Margatoxin/triarylmethane/Phytohemagglutinin	Fluo-3 and FuraRed	2013
Orbán et al. (92)	Human T lymphocytes	Margatoxin/triarylmethane/Phytohemagglutinin	Fluo-3 and FuraRed	2016
Toldi et al. (93)	Human T lymphocytes	Margatoxin/triarylmethane/Phytohemagglutinin	Fluo-3 and FuraRed	2020

SPC, Sphingosylphosphorylcholine.

Regarding the cell compartment analyzed, all the publications obtained by this query referred to the cytosolic Ca^2+^ pool. Among them, 13 articles focused on cytosolic Ca^2+^ levels using the non-ratiometric probes Fluo3 or Fluo4, thus not allowing any resting Ca^2+^ level measurement. The other 8 articles performed ratiometric measurements, mainly through the association of Fluo3 and FuraRed probes: the ratio between the green fluorescence of Fluo3 over the red fluorescence of FuraRed, respectively increasing and decreasing when bound to Ca^2+^, was used as a ratiometric measurement of cytosolic Ca^2+^ level (93, 94). Indeed, ratiometric fluorescent Ca^2+^ indicators minimize the effects of photobleaching, leakage, and uneven loading delivery, allowing more robust and reproducible results. Over the last decades, the combination of Fluo3 and FuraRed has been performed in flow cytometry, displaying a greater response magnitude than the ratiometric probe Indo1 requiring ultraviolet excitation, which is often unavailable on flow cytometers (94). It has been adapted to other cell types, such as platelets (78). Importantly, as FuraRed is a stand-alone ratiometric probe, its single-use showed similar efficiency in ratiometric measurements of cytosolic Ca^2+^ level in PBMCs (95). The single-use of FuraRed, compared to its combination with Fluo3, enables additional cell subtype labeling through additional channels with a faster, cheaper, and more accurate preparation (loading of only one dye).

Regarding the stimulation employed for the Ca^2+^ signaling pathways, the authors focused mainly on extracellular stimulation through the plasma membrane modulation of TCR/BCR or K^+^ channels. Notably, Toldi et al. used flow cytometry to understand how potassium channel inhibition impacts calcium influx in human T lymphocytes of patients with autoimmune disorders, using triarylmethane as a specific inhibitor of IKCa1 channel (44). Flow cytometry also enabled multiparametric analyses to study Ca^2+^ fluxes in T cell subpopulations even in low abundance, i.e., CD4^+^/CD8^+^/CD4^+/^CXCR3^+^ or CD4/CCR4 + (91, 93).

Overall, this query highlights that the study of Ca^2+^ signaling could rely more on flow cytometry. However, the trend is increasing over the last decade (8 out of 21 publications published after 2013). As for the techniques employed, both non-ratiometric and ratiometric probes are used, mainly to assess cytosolic Ca^2+^ levels, therefore putting aside several actors of Ca^2+^ signaling in other cell compartments. From this particular query, only one paper performed intracellular stimulations on a reticular player: Kushnir et al. (17) focused on the major ER Ca^2+^ release channel, ryanodine receptor (RyR), using caffeine as an agonist. They specifically looked at the cytosolic Ca^2+^ level with Fluo4 in human B lymphocytes targeted with an anti-CD19 antibody in PBMCs. The Ca^2+^ response amplitudes obtained by a 50 mM caffeine stimulation were recorded in normal or congestive heart failure lymphocytes and revealed an ER Ca^2+^ leakage as a signature of the pathology.

## 4 Discussion & perspectives

Our literature review query on inflammation and Ca^2+^ signaling revealed 3865 articles published between 1984 and 2022, which supported the design of a bibliometric analysis. We reported that conventional flow cytometry is extensively used in clinical routine as a tool for diagnosis and monitoring of inflammatory diseases. A significant advantage of this method lies in the compensation process to correct fluorescent spillovers, increasing the number of parameters studied and enabling target analysis of specific cell populations. Clinical biomarkers of inflammatory pathologies were identified with this quantitative multi-parametric analysis. More recently, spectral flow cytometry collecting the full light spectrum has enabled the distinction of unique fluorophores with overlapping emission spectra (96, 97). Alongside with technological improvements, ratiometric chemical probes have steadily evolved since the 1980s (26, 95).

**Figure 7:**
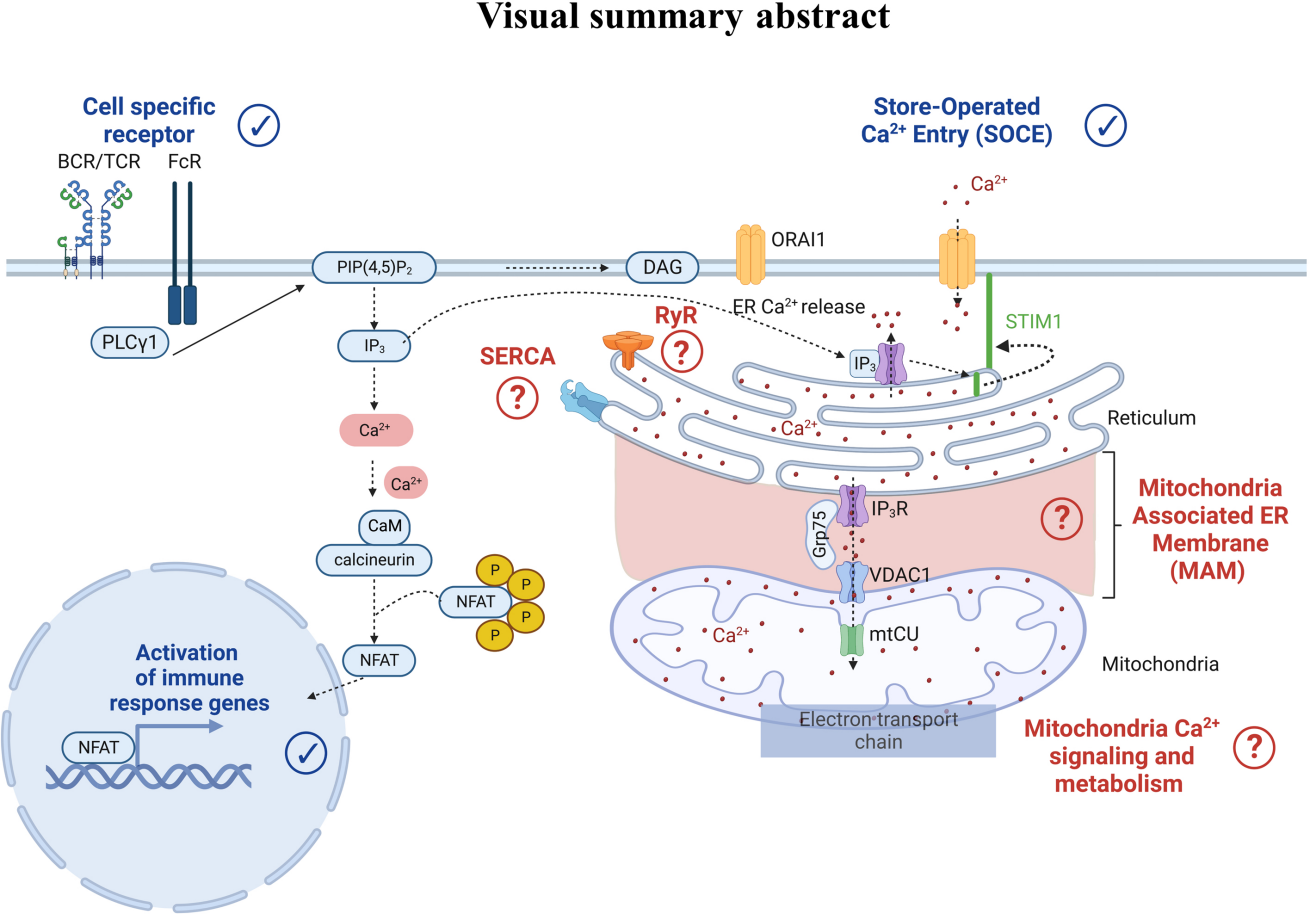
Cell-specific receptor activation through antigens induces protein kinase phosphorylation and phospholipase C gamma 1 activation. The latter leads to the production of the second messenger inositol-1,4,5-triphosphate (IP_3_), which binds to the IP_3_ receptor (IP_3_R) in the endoplasmic reticulum (ER) membrane. IP_3_R activation induces Ca^2+^ efflux from the ER to the cytosol. As a result, an increase of the cytosolic Ca^2+^ concentration and a decrease of the ER Ca^2+^ concentration occur. Sensors in the ER called Stromal Interaction Molecule 1 (STIM1) detect the ER Ca^2+^ decrease, leading to STIM1 oligomerization and translocation to the plasma membrane where it binds to the ORAI1 protein. STIM1-ORA1 interactions contribute to Ca^2+^ influx which elevates the intracellular Ca^2+^ concentration leading to the restoration of the ER Ca^2+^ stocks via repumping through the Sarco-Endoplasmic Reticulum Calcium ATPase (SERCA). In parallel, the increase in cytosolic Ca^2+^ concentration also triggers the calcineurin- NFAT pathways. More recently, Ca^2+^ players such as ryanodine receptors (RyR), SarcoEndoplasmic Reticulum Calcium ATPase (SERCA), the IP3R-Grp75-VDAC complex, and the mitochondrial Ca^2+^ uniporter (mtCU) have emerged as potential contributors to the Ca^2+^ signaling pathway, requiring further dedicated research.

Regarding Ca^2+^ signaling, we highlighted that Ca^2+^ players involved in human PBMC models were studied and described in physiological settings. Indeed, Ca^2+^ ions are ubiquitous intracellular second messengers with a crucial role in the immune cell function: from the cell-specific membrane receptor activation to the SOCE, all contributing to the transcription of immune response genes (**Figure 7**). Therefore, flow cytometry represents a powerful tool compatible with immunophenotyping, allowing the analyses of Ca^2+^ fluxes in immune cell subtypes, even when underrepresented. So far, we have reported only 21 articles using flow cytometry to study Ca^2+^ signaling focused on cytosolic Ca^2+^ level variations bypassing the contribution of organelles, notably the ER as the main Ca^2+^ store and mitochondria whose function depends, in part, on Ca^2+^ signaling. Interestingly, a Ca^2+^ coupling exists between ER and mitochondria to control mitochondrial functions. In this regard, Assis et al. suggested that the width of ER-mitochondria coupling was associated with the macrophage activation status (98). However, whether this structural modification translates into functional consequences on the Ca^2+^ transfer from the ER to mitochondria requires further investigation. Additionally, a recent study demonstrated a reduced mitochondrial Ca^2+^ uptake via the mitochondrial Ca^2+^ uniporter during aging in macrophages as a potential contributor to inflammation in humans (99). The Ryanodine receptor is expressed in immune cells and regulates intracellular Ca^2+^ homeostasis. Osipchuk et al. reported that pharmacological inhibition of RyR leads to intracellular Ca^2+^ alteration and alters immune cell functions *in vitro* and *in vivo* (100). In PBMCs from heart failure patients, Kushnir et al. (17) reported an ER Ca^2+^ leakage in B cells as a biomarker of this pathology. Regarding the SERCA pump, its role is yet to be described in PBMCs. Therefore, the contribution of the different organelles, notably ER, mitochondria, and their coupling, in regulating the immune cell function remains to be deciphered. Albeit combining two Ca^2+^ chemical probes to study two cellular compartments simultaneously remains unexplored. However, with the increasing development of new ratiometric sensors targeting several cell compartments, multiparametric analyses of Ca^2+^ fluxes by flow cytometry may become a promising alternative for human immune cell research in the coming decades.

## Author contributions

CB: Conceptualization, Data curation, Formal Analysis, Investigation, Methodology, Resources, Software, Visualization, Writing – original draft, Writing – review & editing. LC: Conceptualization, Data curation, Formal Analysis, Investigation, Methodology, Software, Writing – review & editing. FM: Conceptualization, Formal Analysis, Investigation, Writing – review & editing. TB: Funding acquisition, Project administration, Supervision, Validation, Writing – review & editing. SD: Conceptualization, Supervision, Validation, Writing – review & editing. MP: Conceptualization, Funding acquisition, Project administration, Supervision, Validation, Writing – review & editing. CCDS: Conceptualization, Funding acquisition, Project administration, Supervision, Validation, Writing – review & editing.
