# Supplementary material for: A bibliometric analysis: Ca2+ fluxes and inflammatory phenotyping by flow cytometry in peripheral blood mononuclear cells
Source: Front Immunol. 2023 Oct 13;14:1272809. doi: 10.3389/fimmu.2023.1272809 (PMC10611513; doi:10.3389/fimmu.2023.1272809)
Supplement: Supplementary file 1 [file DataSheet_1.pdf]

frontiers

Supplementary Material

1 Supplementary Figures

Red Cluster	Lee Gs, 2012	Oppenheim Jj, 1991
Bradford Mm, 1976	Lewis Rs, 2001	Power Ca, 1995
Chomczynski P, 1987	Liou J, 2005	Blue Cluster
Dolmetsch Re, 1997	Mosser Dm, 2008	Choe H, 1996
Dolmetsch Re, 1998	Parekh Ab, 2005	Cocchi F, 1995
Feske S, 2006	Zweifach A, 1993	Deng Hk, 1996
Feske S, 2007	Green Cluster	Doranz Bj, 1996
Franz S, 2011	Baggiolini M, 1994	Dragic T, 1996
Grynkiewicz G, 1985	Charo If, 1994	Yellow Cluster
Hogan Pg, 2010	Gao Jl, 1993	Edgeworth J, 1991
Hoth M, 1992	Murphy Pm, 1994	Odink K, 1987
Laemmli Uk, 1970	Neote K, 1993	Teigelkamp S, 1991

Figure S1: Articles found in the co-citation clusters.

Red Cluster	Steinckwich N, 2015	Sonnenfeld G, 1998	Tiffany H, 1998	Kaufmann A, 2005
Ambudkar I, 2017	Trushin Sa, 2003	Tager A, 2000	Townson J, 2000	La Sala A, 2002
Badou A, 2006	Verheugen J, 1995	Thivierge M, 1999	Vicente-Manzanares M, 1998	Liu L, 2010
Beceiro S, 2017	Wehrhahn J, 2010	Thivierge M, 2006	Vila-Coro A, 2000	Martel-Gallegos G, 2013
Beck A, 2014	Wenning A, 2011	Triggiani M, 2001	Wang J, 1998	Myrtek D, 2008
Beschorner R, 2000	Zhou X, 2014	Ucker Ds, 1992	Wang J, 1999	Pazar M, 2011
Bogeski I, 2010	Zhou Y, 2013	Uehara A, 2002	White J, 2000	Pelegrin P, 2008
Bouhy D, 2011	Zoratti M, 2009	Uehara A, 2003	Wilbanks A, 2001	Skaper S, 2011
Braun A, 2009	Green Cluster	Uehara A, 2004	Wu L, 1997	Stock C, 2006
Braun Mc, 2000	Anel A,1994	Wakita H, 1997	Yanagihara S, 1998	Thomas I, 2010
Carreras-Sureda A, 2013	Aoki Y, 1999	Zocchi E, 1996	Zaitseva M, 1998	Valimaki E, 2016
Chauhan C, 2018	Bader T, 1996	Blue Cluster	Yellow Cluster	Volonte C, 2012
Chen X, 2020	Bozza P, 1996	Burns J, 1999	Al-Aoukaty A, 1998	Wilson Hl, 2004
Cheng Kt, 2012	Carnevale K, 2001	Cheng S, 2001	Allmanniselin I, 1994	Yao H, 2016
Church Ld, 2005	Caronleslie L, 1991	Cole K, 1998	Cai Jp, 1996	Zhong Z, 2013
Deininger Mh, 2000	Chakraborty A, 2007	Crane I, 2000	Campbell Em, 1997	Black Cluster
Demaurex N, 2016	Chang Nca, 2001	Dairaghi Dj, 1998	Charo I, 1994	Combadiere C, 1996
Di L, 2010	Chen Bc, 1998	Damon I, 1998	Chen Bc, 2001	Combadiere C, 1995
Fang K-M, 2011	Clement N, 2006	Delgado E, 1998	Collins Pd, 1993	Garciazepeda Ea, 1996
Felix R, 2013	Coffey M, 1992	Detheux M, 2000	Elsner J, 1997	Hayes Im, 1998
Ferreira R, 2015	Combs Ck, 1999	Elsner J, 2000	Gao Jl, 1993	Heath H, 1997
Fisher W, 2006	Dewin Dr, 2006	Farzan M, 1997	Holmes We, 1991	Imai T, 1997
Gwack Y, 2008	Gottsch Jd, 1999	Franz-Bacon K, 1999	Kaufmann A, 2001	Kitaura M, 1996
Haraguchi K, 2012	Heller T, 1999	Guan E, 2002	Kranzhofer R, 1996	Ponath Pd, 1996
Huang C, 2013	Hsuan Sl, 1999	Guan P, 1999	Lalani As, 1998	Proost P, 1996
Kashio M, 2012	Huang W, 1998	Imai T, 1998	Locati M, 1994	Rothenberg Me, 1996
Khanna R, 1999	Issekutz T, 1992	Isegawa Y,1998	Mccoll Sr, 1993	Sarau Hm, 1997
Kirchhoff K, 2001	Jefferson K, 1999	Liao F, 1999	Miller Md, 1992	Stellato C, 1997
Knowles H, 2013	Kao J, 1994	Martinelli R, 2001	Petering H, 1999	Su Sb, 1996
Lam J, 2011	Kim Y,2004	Mccoll S, 1999	Proost P, 1998	Xu Ll, 1995
Lund F, 2006	Korhonen R, 2001	Menten P, 1999	Salentin R, 2003	Orange Cluster
Masek K, 2006	Kowitz A, 1992	Nagasawa T, 1996	Sanchez X, 1998	Bhardwaj Rs, 1992
Maul-Pavicic A, 2011	Lee Cgl, 1994	Nakano K, 2003	Schraufstatter I, 2004	Eue I, 2000
Mccarl C, 2010	Li X, 2001	Nakano K, 2003	Schutyser E, 2000	Eue I, 2002
Moreno C, 2013	Lotzer K, 2003	Nardelli B, 1999	Shibata F, 2000	Frosch M, 2000
Nam J-H 2009	Madsen P, 1991	Nibbs R, 1997	Struyf S, 1998	Frosch M, 2004
Numaga T, 2010	Mamedova L, 2005	Oh J, 2001	Wang Jm, 1993	Hobbs J, 2003
Ohya S, 2011	Martin L, 2006	Pease J, 1998	Watson Ml, 1998	Hunter Mj, 1998
Ong H, 2012	Mellor E, 2002	Petkovic V, 2004	Wuyts A, 1997	Kerkhoff C, 1998
Panyi G, 2004	Mendez-Samperio P, 2001	Preobrazhensky A, 2000	Wuyts A, 1999	Mahnke K, 1995
Partiseti M, 1994	Mikulski Z, 2010	Princen K, 2003	Wuyts A, 1999	Pagano Rl, 2002
Proescholdt Mg, 2002	Ohmori Y, 1992	Proost P 1998	Zhou D, 1995	Ryckman C, 2003
Sadowska J-M, 2018	Rollet E, 1994	Richardson R, 2000	Purple Cluster	Tan Jq, 1996
Sadowska J-M, 2019	Rosskopf D, 1998	Sabroe I, 1998	Adamczyk M, 2015	Yang Z, 2001
Schappe M, 2018	Sakai A, 2008	Sabroe I, 1999	Brough D, 2003	Brown Cluster
Schluesener Hj, 1998	Sasaki Y, 1993	Schecter A, 2000	Donnelly-Roberts D, 2009	Taguchi K, 2014
Schwab Jm, 2001	Scarfi S, 2008	Schols D, 1997	Hanley P, 2012	Viedt C, 2002
Schwarz A, 2004	Scarfi S, 2009	Struyf S, 1999	Hanley Pj, 2004	
Serafini N, 2012	Schuerermaly Cc, 1994	Struyf S, 2001	Honore P, 2009	
Shideman C.R, 2006	Shinkai A, 1999	Tamaru M, 1998	Hu F, 2015	

Figure S2: Articles within the bibliographic coupling network sorted by cluster.
